# Evaluation of Antibody Responses to COVID-19 Vaccines among Solid Tumor and Hematologic Patients

**DOI:** 10.3390/cancers13174312

**Published:** 2021-08-26

**Authors:** Josef Singer, Nguyen-Son Le, Daniel Mattes, Valerie Klamminger, Klaus Hackner, Nicole Kolinsky, Michaela Scherb, Peter Errhalt, Gudrun Kreye, Martin Pecherstorfer, Sonia Vallet, Klaus Podar

**Affiliations:** 1 Karl Landsteiner University of Health Sciences, Dr. Karl-Dorrek-Straße 30, 3500 Krems, Austria; nguyen-son.le@krems.lknoe.at (N.-S.L.); danielkarl.mattes@krems.lknoe.at (D.M.); valerie.klamminger@krems.lknoe.at (V.K.); klaus.hackner@krems.lknoe.at (K.H.); peter.errhalt@krems.lknoe.at (P.E.); gudrun.kreye@krems.lknoe.at (G.K.); martin.pecherstorfer@krems.lknoe.at (M.P.); sonia.vallet@krems.lknoe.at (S.V.); klaus.podar@krems.lknoe.at (K.P.); 2 Department of Internal Medicine II, University Hospital Krems, Mitterweg 10, 3500 Krems, Austria; nicole.kolinsky@krems.lknoe.at (N.K.); michaela.scherb@krems.lknoe.at (M.S.); 3 Department of Pneumology, University Hospital Krems, Mitterweg 10, 3500 Krems, Austria; 4 Molecular Oncology and Hematology Unit, Karl Landsteiner University of Health Sciences, Dr. Karl-Dorrek-Straße 30, 3500 Krems, Austria

**Keywords:** SARS-CoV-2, COVID-19, SARS-CoV-2 S vaccine, antibody response, cancer patients

## Abstract

**Simple Summary:**

Vaccination is the primary public health strategy to cope with the COVID-19 pandemic. Although solid tumor and hematologic patients are at higher risk of serious COVID-19-related complications and mortality, data on immune responses to COVID-19 vaccines in this patient cohort are particularly scarce. Our results show that antibody titers against the SARS-CoV-2 spike protein are significantly higher in solid tumor vs. hematologic patients. While SARS-CoV-2 antibody titers were equal among sexes, an age-dependent decrease could be observed. Of note, our studies additionally show that complete vaccination represents a valuable predictor for high anti-SARS-CoV-2 antibody responses in solid tumor and hematologic patients. Our findings aim to support future vaccination strategies in these highly vulnerable patients, including vaccination booster programs and alternative protective approaches.

**Abstract:**

Vaccination is the primary public health strategy to cope with the COVID-19 pandemic. Although solid tumor and hematologic patients are at higher risk of serious COVID-19-related complications, data on immune responses to COVID-19 vaccines in this patient cohort are particularly scarce. The present study, therefore, aimed at the standardized determination of anti-SARS-CoV-2 spike protein antibody titers among non-vaccinated versus vaccinated solid tumor and hematologic patients who are under clinical observation or under treatment at the University Hospital Krems. Standardized anti-SARS-CoV-2 S antibody titers of a total of 441 patients were retrospectively analyzed. Our results show that antibody titers against the SARS-CoV-2 spike protein are significantly higher in solid tumor versus hematologic patients. While SARS-CoV-2 antibody titers were equal among sexes, an age-dependent decrease was observed. Of note, our studies additionally show that complete vaccination represents a valuable predictor for high anti-SARS-CoV-2 antibody responses in solid tumor and hematologic patients. In summary, to date, this is one of the largest studies to comprehensively evaluate the impact of various COVID-19 vaccines on anti-SARS-CoV-2 S antibody production in solid tumor and hematologic patients. Our findings aim to support future vaccination strategies in these highly vulnerable patients, including vaccination booster programs and alternative protective approaches.

## 1. Introduction

The coronavirus-disease-19 (COVID-19) pandemic has changed patient care but also everyday life around the world. Due to the high infectivity and morbidity of the Severe Acute Respiratory Syndrome Coronavirus-2 (SARS-CoV-2), high hygiene standards such as wearing face masks in public areas, hand disinfection and social distancing have been established.

Current treatment options include anti-inflammatory agents such as dexamethasone [1] and antiviral drugs, such as remdesivir (Veklury^®^, Gilead Sciences) [2], which may limit severe disease manifestations, but do not prevent primary infection. Thus, vaccination is the primary public health strategy to cope with the COVID-19 pandemic.

Currently, four vaccines against the SARS-CoV-2 spike (S) protein are approved by the European Medical Agencies (EMA): BNT162b2 (Comirnaty^®^, BioNTech/Pfizer), mRNA-1273 (COVID-19 Vaccine Moderna^®^, Moderna), AZD1222 (ChAdOx1, Vaxzevria^®^, AstraZeneca) and JNJ-78436735 (Ad26.COV2.S, COVID-19 Vaccine Janssen^®^, Janssen). Comirnaty^®^ is a lipid nanoparticle-encapsulated mRNA-based SARS-CoV-2 vaccine that encodes the full-length S glycoprotein and is administered in two doses within 19–42 days. Its approval is based on a large (*n* = 43,448) multi-national, placebo-controlled, observer-blinded efficacy study that demonstrated a vaccine-induced 95% protection against the COVID-19 infection. In this trial, only a few tumor patients (3%) were included [3]. COVID-19 Vaccine Moderna^®^ is another mRNA-vaccine, which is administered in two doses within 21–42 days. The approval of this vaccine is based on a large (*n* = 30,420) randomized, observer-blinded, placebo-controlled multicenter phase-three trial conducted in the United States that demonstrated an overall efficacy of 94.1% to prevent (severe) COVID-19 illness. This trial did not report results in hemato-/oncologic patients [4]. Vaxzevria^®^ is a vector-based vaccine to produce the SARS-CoV-2 S glycoprotein antigen. This vaccine is administered in two doses within 28–84 days. Data of four blinded, randomized, controlled trials performed in the United Kingdom, Brazil and South Africa (*n* = 11,636) demonstrated an overall efficacy rate of 70.4% to prevent COVID-19 infections. No data were reported on cancer patients [5]. COVID-19 Vaccine Janssen^®^ is another vector-based vaccine utilizing a recombinant, replication-incompetent adenovirus serotype 26 (Ad26) vector encoding a full-length and stabilized SARS-CoV-2 S protein, which is administered in a single dose. An international, randomized, double-blind, placebo-controlled phase three trial (*n* = 39,321) demonstrated an efficacy rate of this vaccine against severe COVID-19 infections in 76.7% for onset at ≥14 days post-injection and 85.4% for onset at ≥28 days post-injection, respectively. In this trial, participants were excluded with a cancer history ≤1 year before screening [6].

Due to the exciting efficacy rates of these COVID-19 vaccines, vaccination programs were implemented around the world. Health care providers prioritized the elderly and individuals with heart and lung diseases as they are the most vulnerable to COVID-19 infections. Similarly, hemato-/oncologic patients are at higher risk of serious COVID-19 related complications and mortality [7,8,9]. Therefore, the University Hospital Krems initiated a vaccination campaign for hemato-/oncologic patients in spring 2021. Moreover, high hygiene standards and regular COVID-19 antigen- and PCR-tests, as well as the assessment of past infections and anti-SARS-CoV-2 S antibody levels, were implemented as integral parts of the COVID-19 prevention strategy at our University Hospital. Whether the standardized determination of vaccine-induced SARS-CoV-2 S antibody titers is able to inform vaccination strategies in hemato-/oncologic patients, in particular, is mostly unknown. The aim of the present study was to retrospectively analyze COVID-19-vaccine-induced immune responses among solid tumor and hematologic patients at our Institution.

## 2. Materials and Methods

### 2.1. Patients

This retrospective single-center cohort study evaluated oncological (*n* = 269) or hematologic (*n* = 172) patients followed up at the University Hospital Krems. High hygiene standards and regular COVID-19 antigen and PCR tests as well as the assessment of past infections and anti-SARS-CoV-2 S antibody levels were implemented as integral parts of the COVID-19 prevention strategy at our University Hospital. The present study retrospectively analyzed anti-SARS-CoV-2 S antibody levels and data on vaccination or prior infection status deposited at the hospital’s Oncology Information System (OIS) of hemato-/oncologic patients’ records during routine medical visits from 1 March to 31 May 2021. Patients younger than 18 years were excluded. The study was approved by the Institutional Review Board and the Ethics Committee of Lower Austria (GS1-EK-4/696-2021) and was conducted according to the Declaration of Helsinki.

### 2.2. Determination of Antibody Levels

Anti-SARS-CoV-2 S antibody levels were determined in-house from serum of patients utilizing the Elecsys^®^ Anti-SARS-CoV-2 S test system (Roche Diagnostics, Vienna, Austria) and specified as Binding Antibody Units/milliliter (BAU/mL). This quantitative immunoassay for IgG and IgM has a clinical sensitivity of 98.8% (95% CI: 98.1–99.3%) in detecting antibodies reactive to SARS-CoV-2 of patients ≥14 days after a PCR-confirmed COVID-19 infection and a specificity of 100% (95% CI: 99.7–100%) (Elecsys^®^ Anti-SARS-CoV-2 S. Package Insert 2020-09, V1.0; Material Numbers 09289267190 and 09289275190; Roche Diagnostics). The linear range of this test is from 0.4 to 250 BAU/mL. Negative results are thus depicted as 0.39 BAU/mL, and highly reactive samples are capped with a value of 250.01 BAU/mL in this study. Titers >0.8 BAU/mL are considered as reactive. The Elecsys^®^ Anti-SARS-CoV-2 S assay has been compared in 15 clinical samples to a VSV-based pseudo-neutralization assay, which demonstrated that values ≥15 BAU/mL correlate to 92.3% (95% CI 63.97–99.81%) with the presence of neutralizing antibodies [10]. Thus, BAU levels of ≥15/mL were considered as the desired antibody response in our study cohort. Antibody measurements were included from day 10 after the first vaccination. Antibody titers of patients before vaccination or of patients that have declined the vaccination offer served as the “non-vaccinated” control group (*n* = 124). Antibody measurements between day 1 and day 9 after the first vaccination were excluded (*n* = 17). If a patient’s anti-SARS-CoV-2 S antibody levels were determined more than once, the highest value was taken for all analyses, except for the antibody-kinetic analysis. For the latter, all values from day 10 after a first vaccination were included in order to display the kinetics of antibody production as comprehensively as possible. Of note, data of patients vaccinated with COVID-19 Vaccine Janssen^®^ were excluded from the kinetics analysis due to the low patient numbers.

### 2.3. Statistical Analyses

Descriptive statistics include age (median; minimum, maximum values in years), sex, the time of antibody determination in days after the first vaccination (median; minimum, maximum values), the percentage of patients who completed the planned vaccination scheme and the percentage of patients reaching BAU/mL values of ≥15/mL.

Graphs depict median and 95% CI of BAU/mL values.

Normal distribution of continuous variables was assessed with the Shapiro–Wilk test. Correlations were tested by means of *χ*^2^ test.

For multiple group comparisons, Kruskal–Wallis test followed by Dunn’s multiple comparison test was employed. For comparison of two groups, Mann–Whitney test was employed.

Antibody-kinetic graphs show a descriptive plot of BAU/mL on the respective days after the first vaccination.

To identify factors associated with antibody responses, a multivariate linear regression analysis was performed, including ongoing treatment (vs. clinical observation), completion of vaccination (vs. not) and days of antibody analysis from the first vaccination, corrected for sex and age. The *R*-squared and the ANOVA tests were used to assess the adequacy of the models. Multi-collinearity was assessed with variance inflation factors to confirm independence of variables included in the regression model.

Statistical analyses and graphs were performed using IBM SPSS Statistics for Windows v26 (IBM Corp., Armonk, NY, USA) and GraphPad Prism 9.1.2 (GraphPad Software, San Diego, CA, USA). A two-sided significance level of *p* < 0.05 was determined; * indicates *p* < 0.05, ** *p* < 0.01, *** *p* < 0.001 and **** *p* < 0.0001.

## 3. Results

### 3.1. Patient Population

In total, data of 441 patients were included: 215 female, 226 male with a median age of 67 years (years; range: 23–93 years). All patients were followed up at the Departments for Internal Medicine 2 and Pneumology of the University Hospital Krems; 269 patients with solid tumors (137 female, 123 male; median age 65 years, range: 23–90 years) and 172 hematologic patients (78 female, 94 male; median 70 years, range: 25–93 years; Table S1).

### 3.2. SARS-CoV-2 Antibody Levels in Solid Tumor and Hematologic Patients

**Figure 1:**
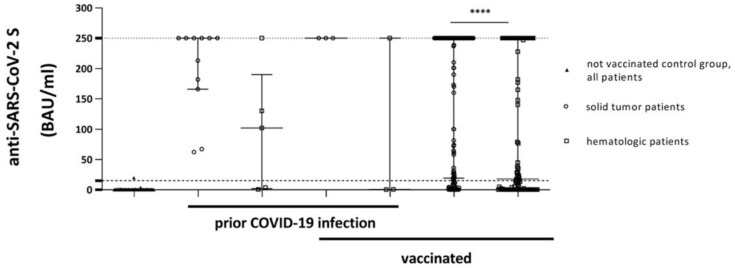
Comparison of antibody levels of included patients: not vaccinated control group, groups with documented prior COVID-19 infections and vaccinated (indexed by thick lines in the description of the x-axis) solid tumor or hematologic patients. Threshold (lower dotted line) indicates ≥15 BAU/mL; the upper dotted line shows the upper limit of the assay (250.01). Displayed are median values and 95% CI of BAU/mL values (upper and lower ends of error bars). BAU: Binding Antibody Units. **** *p* < 0.0001.

Since antibody titers against SARS-CoV-2 S are routinely assessed during medical visits at our Institution, values of many patients were determined before the first vaccination; together, with anti-SARS-CoV-2 S antibody titers of patients who were not vaccinated (e.g., due to a past history of allergic reactions against other vaccines), or who declined vaccination, these values were subsumed into the “non-vaccinated” control group. Out of 124 patients (69 female, 55 male; median age 64 years, range: 23–93 years) of the non-vaccinated control group (median BAU/mL value of 0.39 (95% CI of median: 0.39–0.39)), only two patients displayed a positive reaction with BAU/mL values >0.8. These data indicate a low prevalence of asymptomatic hemato-/oncologic patients with a COVID-19 infection (Figure 1, Table S1). Conversely, these findings further support the higher risk for symptomatic COVID-19 infections in hemato-/oncological patients. Solid tumor patients without vaccination but with a documented history of a past COVID-19 infection (11 patients, 6 female, 5 male; median age 72 years, range: 45–87 years) had median BAU/mL values of 250.0 (95% CI of median: 67.2–250.0). Hematologic patients without vaccination but with a documented past COVID-19 infection (5 patients, 2 female, 3 male; median age 54 years, range: 25–84 years) had lower median BAU/mL values of 102.0 (95% CI of median: 0.56–250.0; Figure 1, Table S1). One hematologic patient did not develop reactive SARS-CoV-2 S antibodies after a documented infection; this patient had received R-CHOP therapy for diffuse large B-cell lymphoma (DLBCL). Another patient developed only moderate levels of reactive antibodies (3.75 BAU/mL); this patient is currently treated with the BTK inhibitor ibrutinib for chronic lymphocytic leukemia (CLL).

At the end of the study period (31 May 2021), 349 patients were vaccinated at least once (79.14%; Vaxzevria^®^: 62, Comirnaty^®^: 147, COVID-19 Vaccine Janssen^®^: 2 and COVID-19 Vaccine Moderna^®^: 138). Vaccinated patients with a past history of COVID-19 infection generally had high BAU/mL values (Figure 1, Table S1). Three out of three solid tumor patients with a past COVID-19 infection and a subsequent vaccination (0 female, 3 male; median age 69 years, range: 28–79 years) had BAU/mL values of 250.1. In contrast, two out of three hematologic patients with a past COVID-19 infection and a subsequent vaccination (2 female, 1 male; median age 82 years, range: 72–85 years) failed to produce reactive antibodies (median BAU/mL value of 0.56 (95% CI of median: 0.39–250.0)) (Figure 1, Table S1). One patient had received obinutuzumab/bendamustine followed by obinutuzumab maintenance therapy for follicular lymphoma (FL); the other patient had received romiplostim for immune thrombocytopenia (ITP). Of note, both patients had completed their vaccination schemes with two doses of mRNA-based vaccines prior to anti-SARS-CoV-2 S antibody determination.

Antibody titers determined ≥10 days after the first vaccination dose in patients without a past history of a COVID-19 infection were included; antibody titers determined before day 10 (nine solid tumor and eight hematologic patients) were excluded from this study. Overall, data of 171 solid tumor patients were analyzed (84 female, 87 male; median age 66 years, range: 28–90 years). The median time of antibody determination after the first vaccination was 50 days (range: 12–122 days). The median BAU/mL value of this cohort was 250.0 (95% CI of median: 250.0–250.0). In contrast, the median BAU/mL value of the hematologic patient cohort (107 patients, 49 female, 58 male; median age 74 years, range: 25–89 years) was 17.9 (95% CI of median: 4.89–76.70). The median time of antibody determination after the first vaccination was 49 days (range 10–130 days; Figure 1, Table S1). Significantly higher antibody levels were obtained in vaccinated solid tumor and vaccinated hematologic patients when compared to the non-vaccinated control group (non-vaccinated vs. vaccinated solid tumor patients: *p* < 0.0001; non-vaccinated vs. vaccinated hematologic patients: *p* < 0.0001; Figure 1). Of note, antibody levels were significantly higher in vaccinated solid tumor patients than in hematologic patients (*p* < 0.0001; Figure 1).

As both vaccinated cohorts were highly heterogeneous, we also determined the percentage of patients that reached a threshold of ≥15 BAU/mL, indicative of the presence of neutralizing antibodies [10]. Anti-SARS-Cov-2 S antibody levels ≥15 BAU/mL were obtained in 134/171 solid tumor (78.4%) patients, but only in 57/107 hematologic (53.3%) patients (*p* < 0.0001). The rate of patients with antibody levels ≥15 BAU/mL was significantly higher in patients who completed the vaccination schema compared to those who did not (86.4% vs. 47.1%, *p* < 0.0001) (Table S1).

**Figure 2:**
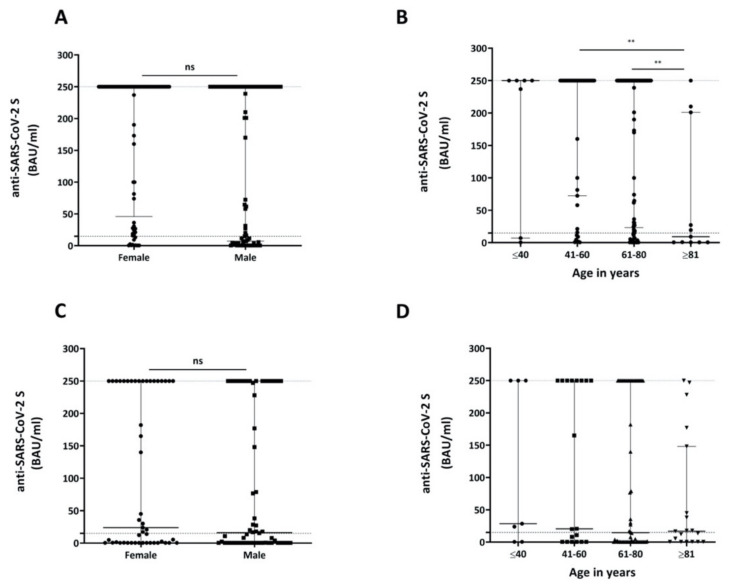
Comparison of antibody levels based on gender or age. (**A**) Comparison of antibody levels after COVID-19 vaccination in solid tumor patients stratified by gender. (**B**) Comparison of different age groups of solid tumor patients with respect to antibody formation upon COVID-19 vaccination. (**C**) Comparison of antibody levels after COVID-19 vaccination in hematologic patients stratified by gender. (**D**) Comparison of different age groups of hematologic therapies with respect to antibody formation upon COVID-19 vaccination. Threshold (lower dotted line) indicates ≥15 BAU/mL; the upper dotted line shows the upper limit of the assay (250.01). Displayed are median values and 95% CI of BAU/mL values (upper and lower ends of error bars). BAU: Binding Antibody Units, ns: not significant; ** *p* < 0.01.

Sex had neither a significant impact on anti-SARS-Cov-2 S antibody production in the solid tumor cohort (Figure 2A, Table S2) nor in the cohort of hematologic patients (Figure 2C, Table S2).

Solid tumor patients ≥81 years of age developed significantly lower levels of antibodies in response to the COVID-19 vaccination than patients aged 41–60 years (*p* = 0.0018) and 61–80 years (*p* = 0.0033; Figure 2B, Table S2). A similar age-dependent effect was also observed among hematologic patients; however, despite a clear trend that could be observed, differences in BAU/mL values among the investigated age groups were not statistically significant (Figure 2D, Table S2).

This study was not designed to compare the effect of different vaccines on antibody formation. However, as the four approved vaccines have different application intervals, a depiction of antibody levels in relation to the days after the first vaccination is shown in Figure S1. These graphs display the different kinetics of antibody formation in solid tumor (Figure S1A) and hematologic patients (Figure S1B). Here, data of patients vaccinated with COVID-19 Vaccine Janssen^®^ are not included due to the low sample size.

### 3.3. Impact of Hemato-/Oncologic Treatment Modalities on COVID-19-Vaccine-Induced Immune Responses

**Figure 3:**
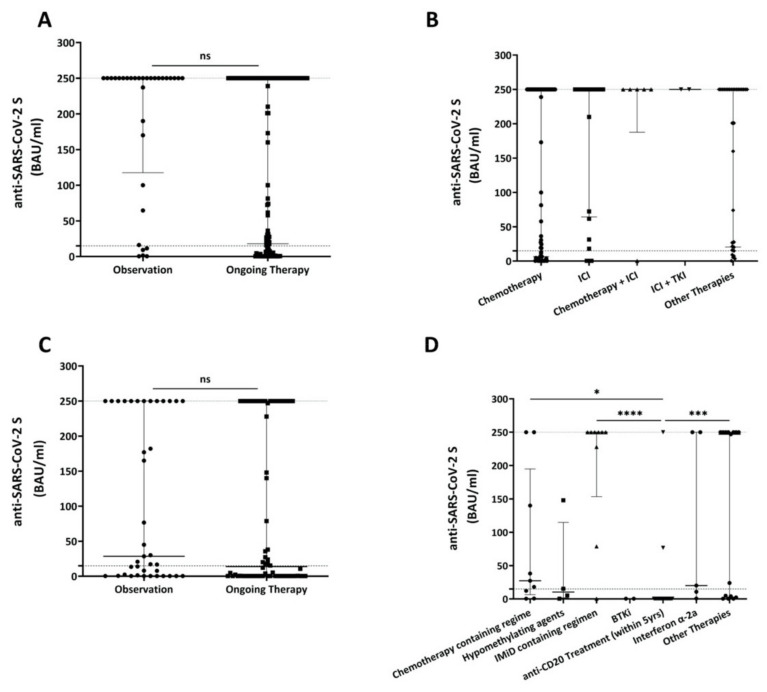
Comparison of antibody levels based on oncological treatment. (**A**) Comparison of antibody levels after COVID-19 vaccination in solid tumor patients that were observed only with patients receiving any type of oncologic therapy. (**B**) Comparison of different oncologic therapies with respect to antibody formation upon COVID-19 vaccination. (**C**) Comparison of antibody levels after COVID-19 vaccination in hematologic patients that were observed only with patients receiving any type of hematologic therapy. (**D**) Comparison of different hematologic therapies with respect to antibody formation upon COVID-19 vaccination. Here, only selected therapies were analyzed. Threshold (lower dotted line) indicates ≥15 BAU/mL; the upper dotted line shows the upper limit of the assay (250.01). Displayed are median values and 95% CI of BAU/mL values (upper and lower ends of error bars). ns: not significant, * *p* < 0.05, *** *p* < 0.001, **** *p* < 0.0001. BAU: Binding Antibody Units; ICI: Immune Checkpoint Inhibitor; TKI:Tyrosine Kinase Inhibitor; IMID: Immunomodulatory Imide Drug; BTKi: Bruton’s Tyrosine Kinase Inhibitor.

We next evaluated the impact of different hemato-/oncologic treatment modalities on COVID-19-vaccine-induced anti-SARS-CoV-2 S antibody production. In the solid tumor patient cohort, no significant difference was observed overall between the clinical observation (i.e., after-care or watch-and-wait) group and the oncologic treatment group (*p* = 0.3139; Figure 3A, Table S3). Subgroup analyses of solid tumor patients suggest that the generation of vaccine-induced anti-SARS-Cov-2 S antibody titers is associated with treatment modalities, with the highest titers in patients treated with immune checkpoint inhibitors alone or in combination with chemotherapy or tyrosine kinase inhibitors (Figure 3B, Table S3).

Similar to solid tumor patients, also in hematologic patients, no significant difference was observed overall between the clinical observation group and the patient group that had received hematologic treatment (*p* = 0.2955; Figure 3C, Table S3). Subgroup analyses showed differences in the production of vaccine-induced anti-SARS-CoV-2 S antibodies upon treatment with distinct modalities, i.e., anti-CD20 monoclonal antibodies in particular (Figure 3D, Table S4). Specifically, to consider the long-term impact of anti-CD20 antibodies on COVID-19-vaccine-induced immune responses, we included all patients who had received anti-CD20 targeting therapies during the last 5 years. Higher anti-SARS-CoV-2 S antibody levels were observed in the subgroup of chemotherapy- or immunomodulatory imide drug (IMiD)-treated patients (median 27.3 BAU/mL, 95% CI of median: 0.65–250; or median 250.0 BAU/mL, 95% CI of median: 78.8–250.0, respectively) than in patients treated with anti-CD20 antibody-containing regimens (ongoing or anytime within the last 5 years); who often showed no antibody response at all (median 0.39, 95% CI of median: 0.39–0.39; *p* = 0.0254). Among anti-CD20 treated patients, only 2 of 24 (8.3%) produced reactive antibodies against SARS-CoV-2 S. One of these patients had received R-CHOP as his last chemo-immunotherapy for mantle cell lymphoma in August 2019, i.e., 19 months prior to his first COVID-19 vaccination with Vaxzevria^®^. Antibody titers in this patient reached a value of 250.01 BAU/mL on day 21 after the first dose. The other patient had received one treatment cycle of rituximab in May 2019 for mantle cell lymphoma, i.e., 23 months prior to his COVID-19 vaccination with Vaccine Moderna^®^. Antibody titers of this patient reached a value of 76.7 BAU/mL on day 18 after the first vaccination.

The low number of patients included warrants additional studies to verify our data on the impact of treatment modalities in hematologic patients in particular (Table S4, Figure 3D).

### 3.4. Impact of Hemato-/Oncologic Diseases on COVID-19-Vaccine-Induced Immune Responses

**Figure 4:**
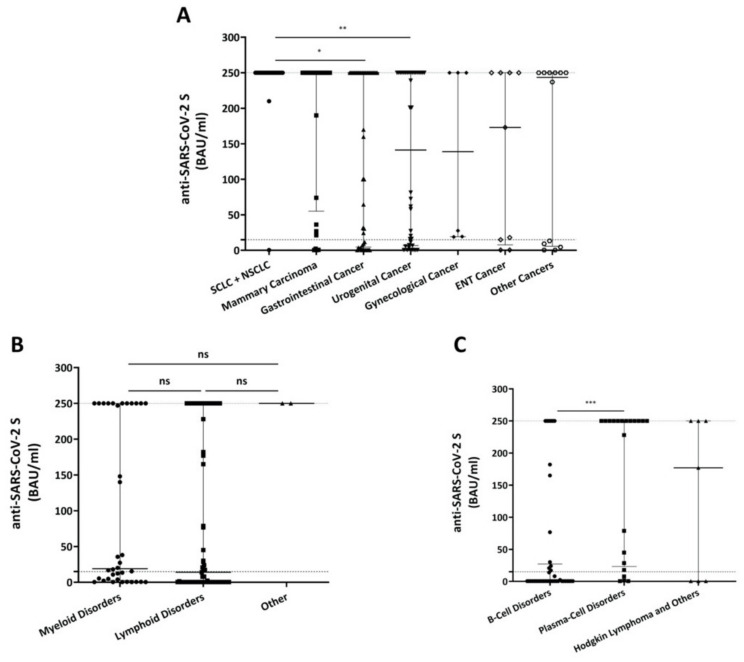
Comparison of antibody levels stratified by type of solid tumor type (**A**) or type of hematologic disease (**B**). (**C**) Comparison of antibody levels after COVID-19 vaccination in hematologic patients with lymphoid diseases grouped by cell of origin of the disease. Threshold (lower dotted line) indicates ≥15 BAU/mL; the upper dotted line shows the upper limit of the assay (250.01). Displayed are median values and 95% CI of BAU/mL values (upper and lower ends of error bars). BAU: Binding Antibody Units, SCLC: Small Cell Lung Cancer, NSCLC: Non-Small Cell Lung Cancer, ENT: Ear-Nose-Throat. ns: not significant, * *p* < 0.05, ** *p* < 0.01, *** *p* < 0.001.

We next investigated whether sub-entities of hemato-/oncologic diseases had an impact on vaccine-induced antibody responses. A high percentage of solid tumor patients achieved the desired antibody levels ≥15 BAU/mL after COVID-19 vaccination (Figure 4A and Table S5). Small cell lung cancer (SCLC), non-small cell lung cancer (NSCLC), as well as gastrointestinal cancer patients presented with significantly higher BAU/mL values (median 250.0, 95% CI of median: 250.0–250.0; median 250.0 BAU/mL, 95% CI of median: 30.6–250.0; *p* = 0.0112), than patients with urogenital malignancies (median 141.2 BAU/mL, 95% CI of median: 16.2–250.0; *p* = 0.0020).

Comparison of patients with hematologic diseases including myeloid, lymphoid (i.e., lymphomas; chronic lymphocytic leukemia, CLL; and immune thrombocytopenia, ITP; multiple myeloma; Morbus Waldenström; Monoclonal Gammopathy of Undetermined Significance, MGUS) and other (i.e., aplastic anemia, hemochromatosis) disorders did not show any significant differences (Figure 4B, Table S5). However, among patients with lymphoid disorders, plasma-cell malignancies produced significantly higher antibody levels (median 141.2 BAU/mL, 95% CI of median: 28.5–250.0) than patients with other B-cell disorders (median 0.39 BAU/mL, 95% CI of median: 0.39–14.0; *p* = 0.0001) (Figure 4C and Table S6).

### 3.5. Multivariate Analysis of Factors Influencing COVID-19-Vaccine-Induced Antibody Responses

Finally, multivariate linear regression analyses were performed, including sex, age, time of antibody testing after the first vaccination, ongoing oncologic treatment vs. observation and completion of vaccination. For solid tumor patients, only younger age and complete vaccination were identified as valuable predictors for high SARS-CoV-2 S antibody response (Table 1). For hematologic patients, younger age was not significant. However, complete vaccination was also a valuable predictor in this cohort (Table 1).

## 4. Discussion

Hemato-/oncologic patients are at higher risk of serious COVID-19 related complications and mortality [7,8,9,11]. Moreover, early reports displayed impaired immunogenicity of the Comirnaty^®^ anti-SARS-CoV-2 vaccine in oncologic patients compared to healthy volunteers [12]. However, data on antibody responses to COVID-19 vaccines in hemato-/oncologic patients are scarce. High hygiene standards, regular COVID-19 antigen and PCR-tests, as well as the assessment of past COVID-19 infections and SARS-CoV-2 S antibody titers, are integral parts of the prevention strategy at our Institution. Whether the determination of standardized vaccine-induced anti-SARS-CoV-2 S antibody titers is able to inform future vaccination strategies in hemato-/oncologic patients in particular is still unclear. We therefore retrospectively analyzed anti-SARS-CoV-2 S titers of 441 patients with solid tumor and hematologic diseases followed up at the Department of Internal Medicine 2 and the Department of Pneumology of the University Hospital Krems between 1 March and 31 May 2021. In the applied Elecsys^®^ Anti-SARS-CoV-2 S test system, values <0.4 BAU/mL are defined as negative, >0.8 BAU/mL are considered as reactive, ≥15 BAU/mL are indicative for the presence of neutralizing antibodies and the upper assay limit is 250 BAU/mL.

Patients treated at our Institution had two opportunities to become vaccinated: (i) by the Nationwide vaccination program of the Federal Republic of Austria or (ii) by the in-house vaccination program. The Nationwide vaccination program utilized all approved COVID-19 vaccines. The latter program, taking place in spring 2021, utilized the COVID-19 Vaccine Moderna^®^ exclusively, which explains its relatively dominant representation in this study. Comirnaty^®^ was the first vaccine to be EMA-approved and delivered in large amounts to Austria. Thus, this vaccine also accounts for a high proportion of vaccinations in our study. It also has the longest antibody kinetics data (Figure S1). Since Vaxzevria^®^ has the longest interval between the two vaccine doses, it represents the vaccine with the highest percentage of patients after just one administration. Of note, the Nationwide vaccination program, which already started in January 2021, prioritized elderly patients and patients living in nursing homes; therefore, longer intervals between the first vaccination and antibody determination as well as higher patient numbers with complete vaccination are included in this subgroup. Our data show that titers against the SARS-CoV-2 S protein are significantly higher in patients with solid tumor vs. hematologic diseases (Figure 1). This is in line with other recently published studies in hematologic patients [13,14], hemato-/oncologic patients [15], patients with multiple myeloma [16] and especially those affected by B-cell disorders, such as CLL [17] or lymphoma [15]. Age is a known risk factor for diminished immune responses to vaccination in general [18]. With regard to anti-SARS-CoV-2 S vaccines, Comirnaty^®^ produced significantly lower antibody levels in individuals >80 years vs. <60 years [19]. Here, we show that higher age was associated with a trend to lower antibody levels in both patient groups. However, despite representing one of the largest cohorts among hemato-/oncological patients, some of our subgroups are very small (solid tumor patients, <40 years: 7; hematologic patients, <40 years: 7; etc.). We therefore chose classification by 20 years’ instead of 10 years’ difference in order to avoid further fragmentation. In the multivariate analysis, the factor age was only significant in the solid tumor patient cohort and predominantly due to the inclusion of patients ≥81 years of age (Figure 2B,D). It is noteworthy that despite all efforts to prioritize this age cohort, only 54.5% of these patients had completed their vaccination scheme during the study period, which adds to the poor outcome of only 45.5% reaching the threshold of ≥15 BAU/mL (Table S2).

Moreover, our analyses also suggest that the generation of vaccine-induced anti-SARS-Cov-2 S antibody titers in hemato-/oncologic patients is associated with treatment modalities, with the highest titers in patients treated with IMiD-containing regimens, and immune checkpoint inhibitors alone or in combination with chemotherapy or tyrosine kinase inhibitors, respectively.

This study is not designed to compare different vaccines, especially because the COVID-19 Vaccine Janssen^®^ was EMA-approved as late as March 2021, and thus only two patients of our cohort received this vaccine and were tested for antibodies subsequently. Nonetheless, our data on the kinetics of antibody production in days after the first vaccination suggest that anti-SARS-CoV-2 S antibody levels are higher in solid tumor patients, also at shorter time points post-injection. Moreover, the percentage of solid tumor patients that fail to produce anti-SARS-CoV-2 S antibodies is lower when compared to hematologic patients. Patients who have received an mRNA-based vaccine (Comirnaty^®^ or COVID-19 Vaccine Moderna^®^) presented with higher BAU/mL values, at earlier time points, than patients who have received Vaxzevria^®^. However, since the second dose of Vaxzevria^®^ was administered as late as 84 days after the first dose, our observation period is too short to accurately display the kinetics of Vaxzevria^®^-induced antibodies (Figure S1).

In order to determine if and when a booster injection should be administered, the durability of vaccine-induced antibody responses represents one, if not the most important unanswered question of current COVID-19 management. As the presence of neutralizing antibodies against SARS-CoV-2 S highly correlates with ≥15 BAU/mL levels, we defined this level as the desired threshold for successful vaccinations in our study. Of interest, our analyses suggest that solid tumor as well as hematologic patients maintain anti-SARS-CoV-2 S antibody levels above this threshold even after 120 days ensuing the first vaccination. However, the observation period of our study is too short for a final conclusion. Therefore, larger follow-up studies are needed with longer observation periods.

All vaccines utilized in this study induce specific T-cell responses [4,5,20,21]. Moreover, a recent case series showed that a third vaccine boost increased anti-SARS-CoV-2 S titers in organ transplant recipients with a minimal/no antibody response after two vaccine doses [22]. The high relevance of vaccine-induced cellular responses in anti-SARS-CoV-2 S induced immune responses is supported by uncomplicated courses of the COVID-19 disease in patients with agammaglobulinemia [21], which is characterized by the complete absence of B-cells. Large prospective clinical trials are therefore needed that also take into account vaccine-induced cellular responses, on CD4+ and CD8+ T-cells in particular, to gain a complete picture of all vaccine-induced protective immunological effects. Based on our present and other data, these investigations are especially needed in hematologic patients diagnosed with B-cell disorders and/or treated with CD20 targeting therapies.

Considering CD20 targeting, promising data have recently been shown by Mrak et al., where a proportion of patients suffering from rheumatic diseases treated with rituximab displayed anti-SARS-CoV-2 S antibodies after vaccination with mRNA-based SARS-CoV-2 vaccines, once peripheral B-cells at least partially repopulate [23].

Whether complete vaccination is essential for the development of an adequate immune response in the general population, and hemato-/oncologic patients in particular, represents another, if not the most, relevant unanswered question in current public health discussions. Importantly, data presented in this study underscore the pre-eminent role of complete vaccination schemes for all COVID-19 approved vaccines for the production of maximal antibody titers among our patient cohorts. These data are also supported by recent findings, which stress the importance of administering both doses of mRNA-based [24] and also vector-based anti-SARS-CoV-2 S vaccines in cancer patients [25]. Taken together, accumulating evidence strongly supports the need for complete vaccination for hemato-/oncologic patients, especially in the light of upcoming new variants of SARS-CoV-2 S.

### Limitations

An important limitation of this study is that antibody responses of patients that exceeded the linear range of the Elecsys^®^ Anti-SARS-CoV-2 S test are capped with a value of 250.01 BAU/mL in these analyses. By that, important information is omitted: first, it would be interesting to know to what extent antibody levels against SARS-CoV-2 can be achieved in distinct patient groups, and second, this further discrimination would help in obtaining a better understanding of the kinetics of antibody responses over time. By knowing the highest achieved levels, timepoints for eventual booster vaccinations could be better extrapolated. We thus suggest for future studies to determine exact values of BAU/mL; if values exceed the linear range of the Elecsys^®^ Anti-SARS-CoV-2 S test, a 1:10 dilution of the sample can be measured subsequently. This is also recommended in the package insert of the test.

Moreover, only humoral immune responses to SARS-CoV-2 vaccines were measured. Future studies should also include information on the extent and durability of elicited cellular immune responses.

## 5. Conclusions

In summary, to date, this is one of the largest studies to comprehensively evaluate the impact of various COVID-19 vaccines on SARS-CoV-2 S antibody production in solid tumor and hematologic patients. Results aim to inform future vaccination strategies in these highly vulnerable patients, including vaccination booster programs and alternative protective approaches.

## Figures and Tables

**Table 1:** Variables associated with antibody response after SARS-COV-2 vaccination in patients with solid tumor (*n* = 171) and hematologic diseases (*n* = 107). Multivariate linear regression model included completion of vaccination scheme, day of Antibody testing after first vaccine and oncological treatment (ongoing vs. observation-only) and was corrected for sex and age.

Patient Groups	Factors	Unstandardized Coefficients Beta	95.0% CI		*p*-Value	Adjusted *R*^2^	*F* Value
			Lower Bound	Upper Bound			
**Vaccinated solid tumor patients**	Sex	20.352	−3.252	43.957	0.091	0.53	39.9
	Age	−1.561	−2.569	−0.552	**0.003**	-	-
	Completion of vaccination scheme	170.454	138.855	202.054	**0.000**	-	-
	Day of Ab testing after first vaccine	0.453	−0.189	1.094	0.165	-	-
	Ongoing oncological treatment	−26.922	−56.133	2.289	0.071	-	-
**Vaccinated hematologic patients**	Sex	26.870	15.420	69.159	0.210	0.13	4.2
	Age	−1.328	−2.826	0.169	0.082	-	-
	Completion of vaccination scheme	106.050	48.891	163.209	**0.000**	-	-
	Day of Ab testing after first vaccine	−0.140	−1.264	0.984	0.805	-	-
	Ongoing hematological treatment	31.434	75.663	12.796	0.162	-	-

Significant *p*-values are displayed bold. Ab: Antibody; CI: Confidence Interval.

## Data Availability

Data remain at the University Hospital Krems. The datasets used and analyzed during the current study are available from the corresponding author on reasonable request.

## Supplementary Materials

The following are available online at https://www.mdpi.com/article/10.3390/cancers13174312/s1, Figure S1: Kinetics of antibody formation displayed in days after first COVID-19 vaccination, Table S1: Patient characteristics and BAU/mL values of all included patients, non-vaccinated control group, groups with documented prior COVID-19 infections and vaccinated solid tumor or hematologic patients, respectively, Table S2: Patient characteristics and BAU/mL values of vaccinated solid tumor or hematologic patients, grouped by gender or age, Table S3: Patient characteristics and BAU/mL values of vaccinated solid tumor or hematologic patients, grouped therapy status, Table S4: Patient characteristics and BAU/mL values of vaccinated solid tumor or hematologic patients, grouped by different oncologic treatment, Table S5: Patient characteristics and BAU/mL values of solid tumor or hematologic patients, grouped by type of disease, Table S6: Patient characteristics and BAU/mL values of all vaccinated hematologic patients with lymphoid disorders, grouped by cell of origin of the disease.

## Abbreviations

BAU/mL	Binding Antibody Units/Milliliter
BTKi	Bruton’s Tyrosine Kinase Inhibitor
CI	Confidence Interval
CLL	Chronic Lymphocytic Leukemia
COVID-19	Coronavirus-Disease-19
DLBCL	Diffuse Large B-Cell Lymphoma
EMA	European Medical Agencies
ENT	Ear–Nose–Throat
ICI	Immune Checkpoint Inhibitor
IMiD	Immunomodulatory Imide Drug
ITP	Immunthrombocytopenia
R-CHOP	Rituximab, Cyclophosphamide, Hydroxydaunorubicin, Oncovin, Prednisolone
SARS-CoV-2	Severe Acute Respiratory Syndrome Coronavirus-2
SCLC	Small Cell Lung Cancer
NSCLC	Non-Small Cell Lung Cancer
TKI	Tyrosine Kinase Inhibitor

## References

[1] Group R.C., Horby P., Lim W.S., Emberson J.R., Mafham M., Bell J.L., Linsell L., Staplin N., Brightling C., Ustianowski A. (2021). Dexamethasone in Hospitalized Patients with Covid-19. N. Engl. J. Med..

[2] Beigel J.H., Tomashek K.M., Dodd L.E., Mehta A.K., Zingman B.S., Kalil A.C., Hohmann E., Chu H.Y., Luetkemeyer A., Kline S. (2020). Remdesivir for the treatment of Covid-19—Final report. N. Engl. J. Med..

[3] Polack F.P., Thomas S.J., Kitchin N., Absalon J., Gurtman A., Lockhart S., Perez J.L., Perez Marc G., Moreira E.D., Zerbini C. (2020). Safety and efficacy of the BNT162b2 mRNA Covid-19 vaccine. N. Engl. J. Med..

[4] Baden L.R., El Sahly H.M., Essink B., Kotloff K., Frey S., Novak R., Diemert D., Spector S.A., Rouphael N., Creech C.B. (2021). Efficacy and Safety of the mRNA-1273 SARS-CoV-2 Vaccine. N. Engl. J. Med..

[5] Voysey M., Clemens S.A.C., Madhi S.A., Weckx L.Y., Folegatti P.M., Aley P.K., Angus B., Baillie V.L., Barnabas S.L., Bhorat Q.E. (2021). Safety and efficacy of the ChAdOx1 nCoV-19 vaccine (AZD1222) against SARS-CoV-2: An interim analysis of four randomised controlled trials in Brazil, South Africa, and the UK. Lancet.

[6] Sadoff J., Gray G., Vandebosch A., Cardenas V., Shukarev G., Grinsztejn B., Goepfert P.A., Truyers C., Fennema H., Spiessens B. (2021). Safety and efficacy of single-dose Ad26.COV2.S vaccine against Covid-19. N. Engl. J. Med..

[7] Bakouny Z., Hawley J.E., Choueiri T.K., Peters S., Rini B.I., Warner J.L., Painter C.A. (2020). COVID-19 and cancer: Current challenges and perspectives. Cancer Cell.

[8] Grivas P., Khaki A.R., Wise-Draper T.M., French B., Hennessy C., Hsu C.Y., Shyr Y., Li X., Choueiri T.K., Painter C.A. (2021). Association of clinical factors and recent anticancer therapy with COVID-19 severity among patients with cancer: A report from the COVID-19 and Cancer Consortium. Ann. Oncol..

[9] Kuderer N.M., Choueiri T.K., Shah D.P., Shyr Y., Rubinstein S.M., Rivera D.R., Shete S., Hsu C.Y., Desai A., de Lima Lopes G. (2020). Clinical impact of COVID-19 on patients with cancer (CCC19): A cohort study. Lancet.

[10] Meyer B., Torriani G., Yerly S., Mazza L., Calame A., Arm-Vernez I., Zimmer G., Agoritsas T., Stirnemann J., Spechbach H. (2020). Validation of a commercially available SARS-CoV-2 serological immunoassay. Clin. Microbiol. Infect..

[11] Assaad S., Avrillon V., Fournier M.L., Mastroianni B., Russias B., Swalduz A., Cassier P., Eberst L., Steineur M.P., Kazes M. (2020). High mortality rate in cancer patients with symptoms of COVID-19 with or without detectable SARS-COV-2 on RT-PCR. Eur. J. Cancer.

[12] Barriere J., Chamorey E., Adjtoutah Z., Castelnau O., Mahamat A., Marco S., Petit E., Leysalle A., Raimondi V., Carles M. (2021). Impaired immunogenicity of BNT162b2 anti-SARS-CoV-2 vaccine in patients treated for solid tumors. Ann. Oncol..

[13] Re D., Barriere J., Chamorey E., Delforge M., Gastaud L., Petit E., Chaminade A., Verriere B., Peyrade F. (2021). Low rate of seroconversion after mRNA anti-SARS-CoV-2 vaccination in patients with hematological malignancies. Leuk. Lymphoma.

[14] Maneikis K., Sablauskas K., Ringeleviciute U., Vaitekenaite V., Cekauskiene R., Kryzauskaite L., Naumovas D., Banys V., Peceliunas V., Beinortas T. (2021). Immunogenicity of the BNT162b2 COVID-19 mRNA vaccine and early clinical outcomes in patients with haematological malignancies in Lithuania: A national prospective cohort study. Lancet Haematol..

[15] Benda M., Mutschlechner B., Ulmer H., Grabher C., Severgnini L., Volgger A., Reimann P., Lang T., Atzl M., Huynh M. (2021). Serological SARS-CoV-2 antibody response, potential predictive markers and safety of BNT162b2 mRNA COVID-19 vaccine in haematological and oncological patients. Br. J. Haematol..

[16] Ghandili S., Schonlein M., Lutgehetmann M., Schulze Zur Wiesch J., Becher H., Bokemeyer C., Sinn M., Weisel K.C., Leypoldt L.B. (2021). Post-vaccination anti-SARS-CoV-2-antibody response in patients with multiple myeloma correlates with low CD19+ B-lymphocyte count and anti-cd38 treatment. Cancers.

[17] Roeker L.E., Knorr D.A., Thompson M.C., Nivar M., Lebowitz S., Peters N., Deonarine I., Momotaj S., Sharan S., Chanlatte V. (2021). COVID-19 vaccine efficacy in patients with chronic lymphocytic leukemia. Leukemia.

[18] Sadighi Akha A.A. (2018). Aging and the immune system: An overview. J. Immunol. Methods.

[19] Muller L., Andree M., Moskorz W., Drexler I., Walotka L., Grothmann R., Ptok J., Hillebrandt J., Ritchie A., Rabl D. (2021). Age-dependent immune response to the Biontech/Pfizer BNT162b2 COVID-19 vaccination. Clin. Infect. Dis..

[20] Sadoff J., Le Gars M., Shukarev G., Heerwegh D., Truyers C., de Groot A.M., Stoop J., Tete S., Van Damme W., Leroux-Roels I. (2021). Interim results of a phase 1-2a trial of Ad26.COV2.S Covid-19 vaccine. N. Engl. J. Med..

[21] Tarke A., Sidney J., Methot N., Yu E.D., Zhang Y., Dan J.M., Goodwin B., Rubiro P., Sutherland A., Wang E. (2021). Impact of SARS-CoV-2 variants on the total CD4(+) and CD8(+) T cell reactivity in infected or vaccinated individuals. Cell. Rep. Med..

[22] Werbel W.A., Boyarsky B.J., Ou M.T., Massie A.B., Tobian A.A.R., Garonzik-Wang J.M., Segev D.L. (2021). Safety and immunogenicity of a third dose of SARS-CoV-2 vaccine in solid organ transplant recipients: A case series. Ann. Intern. Med..

[23] Mrak D., Tobudic S., Koblischke M., Graninger M., Radner H., Sieghart D., Hofer P., Perkmann T., Haslacher H., Thalhammer R. (2021). SARS-CoV-2 vaccination in rituximab-treated patients: B cells promote humoral immune responses in the presence of T-cell-mediated immunity. Ann. Rheum. Dis..

[24] Addeo A., Shah P.K., Bordry N., Hudson R.D., Albracht B., Di Marco M., Kaklamani V., Dietrich P.Y., Taylor B.S., Simand P.F. (2021). Immunogenicity of SARS-CoV-2 messenger RNA vaccines in patients with cancer. Cancer Cell.

[25] Heudel P., Favier B., Assaad S., Zrounba P., Blay J.Y. (2021). Reduced SARS-COV-2 infection and death after two doses of COViD-19 vaccines in a series of 1503 cancer patients. Ann. Oncol..

