# Supplementary material for: Evaluation of Antibody Responses to COVID-19 Vaccines among Solid Tumor and Hematologic Patients
Source: Cancers (Basel). 2021 Aug 26;13(17):4312. doi: 10.3390/cancers13174312 (PMC8430869; doi:10.3390/cancers13174312)
Supplement: Supplementary file 1 [file cancers-13-04312-s001.zip › cancers-1335667-supplementary.pdf]

Supplementary Materials

Evaluation of Antibody Responses to COVID-19 Vaccines Among Solid Tumor and Hematologic Patients.

Josef Singer, Nguyen-Son Le, Daniel Mattes, Valerie Klamminger, Klaus Hackner, Nicole Kolinsky, Michaela Scherb, Peter Errhalt, Gudrun Kreye, Martin Pecherstorfer, Sonia Vallet and Klaus Podar

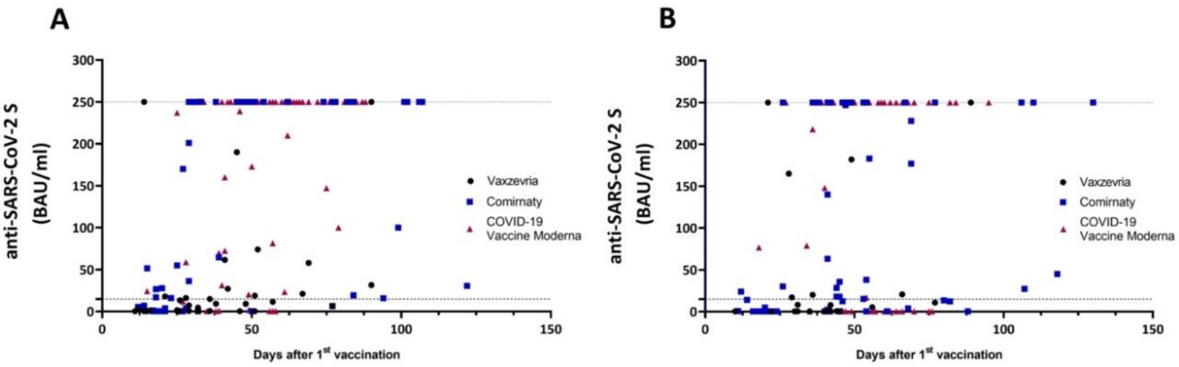

Figure S1. Kinetics of antibody formation displayed in days after first COVID-19 vaccination. (**A**) solid tumor patients; (**B**) hematologic patients. BAU/mL values of patients vaccinated with Vaxzevria are shown as black circles; patients vaccinated with Comirnaty are displayed as blue squares and antibody levels of patients vaccinated with COVID-10 vaccine Moderna are depicted as red triangles. Threshold (lower dotted line) indicates ≥15 BAU/mL; the upper dotted line shows the upper limit of the assay (250.01 BAU/mL).

Table S1. Patient characteristics and BAU/mL values of all included patients, not vaccinated control group, groups with documented prior COVID-19 infections and vaccinated solid tumor or hematologic patients, respectively.

	Patient Characteristics (Age: median; minimum - maximum)	Vaccine Type (Vaxzevria®/ Comirnaty®/ COVID-19 Vaccine Janssen®/ COVID-19 Vaccine Moderna®)	Ab-analysis after first vaccination (median; minimum-maximum)	Vaccination Scheme completed	Ab-levels ≥ 15 BAU/ml	BAU/ml Median (95% CI of median)
**Included patients (total)**	441: 215 female, 226 male (Age: 67yrs; 23yrs-93yrs)					
	Solid tumor patients: 269 (137 female, 123 male; 65yrs; 23-90yrs)					
	Hematologic patients: 172 (78 female, 94 male; 70yrs; 25-93yrs)					
**Patients without vaccination or prior documented COVID-Infection**	124: 69 female, 55 male (64yrs; 23-93yrs) 75 solid tumor patients 49 hematologic patients	not applicable	not applicable	0%	0.8%	0.39 (0.39-0.39)
**Patients without vaccination but prior documented COVID-Infection**	16					
	11 solid tumor patients 6 female, 5 male (72yrs; 45-87yrs)	not applicable	not applicable	0%	100%	250.0 (67.2-250.0)
	5 hematologic patients 2 female, 3 male (54yrs; 25-84yrs)	not applicable	not applicable	0%	60%	102.0 (0.56 – 250.0)
**Patients with vaccination plus prior documented COVID-Infection**	6					
	3 solid tumor patients 0 female, 3 male (69yrs; 28-79yrs)	1/2/0/0	not applicable	16.7%	100%	250.0 (250.0-250.0)
	3 hematologic patients 2 female, 1 male (82yrs; 72-85yrs)	0/3/0/0	not applicable	66.6%	33.3%	0.56 (0.39-250.0)
**Vaccinated solid tumor patients**	171					
	84 female, 87 male (66yrs, 28-90yrs)	29/46/1/95	50d; 12-122d	76%	78.4%	250.0 (250.0-250.0)
**Vaccinated hematologic patients**	107					
	49 female, 58 male (74yrs, 25-89yrs)	21/44/0/42	49d; 10-130d	72.9%	53.3%	17.9 (4.89-76.70)

Yrs: years; BAU/ml: Binding Antibody Units/Milliliter; CI: Confidence Interval.

Table S2. Patient characteristics and BAU/mL values of vaccinated solid tumor or hematologic patients, grouped by gender or age.

	Patient Characteristics (Age: Median; Minimum –Maximum)	Vaccine Type (Vaxzevria®/Comirnaty®/COVID-19 Vaccine Janssen®/COVID-19 Vaccine Moderna®)	Ab-Analysis After First Vaccination (Median; Minimum–Maximum)	Vaccination Scheme Completed	Ab-Levels ≥15 BAU/mL	BAU/mL Median (95% CI of Median)
**Vaccinated solid tumor patients**	**Gender:**					
	Female 84 (64 yrs; 33–85 yrs)	14/22/0/48	53d; 12–122d	78.6%	86.9%	250.0 (250.0–250.0)
	Male 87 (68 yrs; 28–90 yrs)	15/24/1/47	46d; 12–87d	73.6%	70.1%	250.0 (64.6–250.0)
	**Age:**					
	≤40 years 7: 3 female, 4 male (33 yrs; 28–40 yrs)	1/4/1/1	25d; 14–122d	57.1%	71.4%	250.0 (0.39–250.0)
	41–60 years 43: 26 female, 17 male (56 yrs; 42–60 yrs)	9/6/0/28	56d; 16–106d	79.1%	83.7%	250.0 (250.0–250.0)
	61–80 years 110: 52 female, 58 male (69 yrs; 61–80 yrs)	15/32/0/63	49d; 12–122d	78.2%	80%	250.0 (250.0–250.0)
	≥81 years 11: 3 female, 8 male (84 yrs; 81–90 yrs)	4/4/0/3	38d; 19–84d	54.5%	45.5%	9.32 (0.39–210.0)
**Vaccinated hematologic patients**	**Gender:**					
	Female 49: (73 yrs; 31–89 yrs)	13/19/0/17	49d; 10–130d	67.4%	55.1%	24.0 (2.41–250.0)
	Male 58: (75 yrs; 25–85 yrs)	8/25/0/24	49d; 11–107d	72.9%	51.7%	16.05 (0.66–76.7)
	**Age:**					
	≤40 years 7: 4 female, 3 male (37 yrs; 25–40 yrs)	0/6/0/1	56d; 12–130d	57.1%	71.4%	28.5 (0.39–250.0)
	41–60 years 19: 8 female, 11 male (53 yrs; 45–60 yrs)	8/3/0/8	50d; 28–106d	68.4%	57.9%	20.7 (0.39–250.0)
	61–80 years 62: 31 female, 31 male (75 yrs; 62–80 yrs)	9/24/0/29	46d; 11–107d	74.2%	50%	14.65 (0.66–182.0)
	≥81 years 19: 6 female, 13 male (83 yrs; 81–89 yrs)	4/11/0/4	54d; 10–118d	78.9%	52.6%	16.8 (0.39–148.0)

Yrs: years; BAU/ml: Binding Antibody Units/Milliliter; CI: Confidence Interval.

Table S3. Patient characteristics and BAU/mL values of vaccinated solid tumor or hematologic patients, grouped by therapy status.

	Patient Characteristics (Age: Median; Minimum–Maximum)	Vaccine Type (Vaxzevria®/Comirnaty®/ COVID-19 Vaccine Janssen®/COVID-19 Vaccine Moderna®)	Ab-Analysis After First Vaccination (Median; Minimum–Maximum)	Vaccination Scheme Completed	Ab-Levels ≥15 BAU/mL	BAU/mL Median (95% CI of Median)
**Vaccinated solid tumor patients**	*Observation only* 32: 16 female, 16 male (70 yrs; 28–85 yrs)	7/16/0/9	47d; 14–107d	71.9%	87.5%	250.0 (190.0–250.0)
	Ongoing therapy 139: 68 female, 71 male (65 yrs; 33–90 yrs)	22/30/1/86	50d; 12–122d	7%	76.3%	250.0 (239.0–250.0)
**Vaccinated hematologic patients**	*Observation only* 39: 22 female, 17 male (72 yrs, 25–88 yrs)	10/14/0/15	49d; 11–118d	64.1%	59%	28.5 (7.82–250.0)
	Ongoing therapy 68: 27 female, 41 male (76 yrs, 31–89 yrs)	11/30/0/27	49d; 10–130d	77.9%	50%	13.8 (0.4–78.8)

Yrs: years; BAU/ml: Binding Antibody Units/Milliliter; CI: Confidence Interval.

Table S4. Patient characteristics and BAU/mL values of vaccinated solid tumor or hematologic patients, grouped by different oncologic treatments.

	Patient Characteristics (Age: Median; Minimum–Maximum)	Vaccine Type (Vaxzevria®/Comirnaty®/ COVID-19 Vaccine Janssen®/COVID-19 Vaccine Moderna®)	Ab-Analysis After First Vaccination (Median; Minimum–Maximum)	Vaccination Scheme Completed	Ab-Levels ≥15 BAU/mL	BAU/mL Median (95% CI of median)
**Vaccinated solid tumor patients**	*Chemotherapy (alone, + targeted therapy, + hormonal therapy, + immunotherapies other than ICI)* 71: 42 female, 29 male (66 yrs; 33–90 yrs)	12/15/1/43	50d; 12–122d	74.7%	70.4%	250.0 (36.3–250.0)
	ICI alone or ICI + ICI 28: 8 female, 20 male (65 yrs; 52–84 yrs)	4/2/0/22	53d; 21–90d	85.7%	85.7%	250.0 (210.0–250.0)
	Chemotherapy + ICI 6: 3 female, 3 male (64 yrs; 52–80 yrs)	0/1/0/5	56d; 32–62d	100%	83.3%	250.0 (0.39–250.0)
	ICI + TKI 2: 0 female, 2 male (64 yrs, 75 yrs)	0/1/0/1	55d; 32–77d	50%	100%	250.0 (250.0–250.0)
	Other therapies (TKI, Hormonal therapy, Radiation) 32: 18 female, 14 male (66 yrs; 40–82 yrs)	6/11/0/15	48d; 12–88d	71.9%	78.1%	250.0 (26.8–250.0)
**Vaccinated hematologic patients**	*Chemotherapy-containing regimens* 9: 4 female, 5 male (79 yrs; 45–87 yrs)	2/7/0/0	49d; 31–107d	77.8%	66.7%	27.3 (0.65–250.0)
	Hypomethylating agents 4: 1 female, 3 male (83 yrs; 69–89 yrs)	1/2/0/1	47d; 40–56d	75%	50%	10.36 (0.39–148.0)
	IMiD-containing regimens 9: 5 female, 4 male (72 yrs; 57–86 yrs)	1/1/0/7	50d; 34–69d	77.8%	88.9%	250.0 (78.8–250.0)
	BTKi treatment 2: 1 female, 1 male (67 yrs; 76 yrs)	0/0/0/2	62d; 60–64d	100%	0%	0.39 (0.39–0.39)
	Anti-CD20 treatment within the last 5 years 24: 8 female, 16 male (75 yrs, 39–88 yrs)	4/5/0/15	55d; 10–88d	79.2%	8.3%	0.39 (0.39–0.39)
	Interferon α-2a 5: 3 female, 2 male (46 yrs; 37–75 yrs)	3/2/0/0	67d; 36–72d	40%	0%	20.1 (0.72–250.0)
	Other therapies (Anagrelid, Ciclosporin, Imatinib, Prednisolon, Romiplostim, Phlebotomy) 15: 5 female, 10 male (75 yrs, 31–83 yrs)	0/13/0/2	36d; 12–82d	66.7%	60%	247.0 (2.23–250.0)

Yrs: years; BAU/ml: Binding Antibody Units/Milliliter; CI: Confidence Interval; ICI: Immune Checkpoint Inhibitor; TKI: Tyrosine Kinase Inhibitor; IMiD: Immunomodulatory Imide Drug; BTKi: Bruton's Tyrosine Kinase Inhibitor.

Table S5. Patient characteristics and BAU/mL values of solid tumor or hematologic patients, grouped by type of disease.

	Patient Characteristics (Age: Median; Minimum–Maximum)	Vaccine Type (Vaxzevria®/Comirnaty®/ COVID-19 Vaccine Janssen®/COVID-19 Vaccine Moderna®)	Ab-Analysis After First Vaccination (Median; Minimum–Maximum)	Vaccination Scheme Completed	Ab-Levels ≥15 BAU/mL	BAU/mL Median (95% CI of Median)
**Vaccinated solid tumor patients**	*SCLC + NSCLC Patients*<br>28: 10 female, 18 male<br>(62 yrs; 42–84 yrs)<br>0 observation , 28 ongoing therapy	1/1/0/26	58d; 32–90d	100%	96.4%	250.0 (250.0–250.0)
	Mammary Carcinoma Patients<br>29: 28 female, 1 male<br>(64 yrs; 43–80 yrs)<br>3 observation, 26 ongoing therapy	5/9/0/15	51d; 12–106d	79.3%	86.2%	250.0 (190.0–250.0)
	Gastrointestinal Cancer Patients<br>48: 22 female, 26 male<br>(71 yrs; 38–90 yrs)<br>12 observation, 36 ongoing therapy	6/17/0/25	48d; 14–122d	75%	70.8%	250.0 (30.6–250.0)
	Urogenital Cancer Patients<br>40: 7 female, 33 male<br>(68 yrs; 28–86 yrs)<br>8 observation, 32 ongoing therapy	10/11/1/18	42d; 12–102d	65%	70%	141.2 (16.2–250.0)
	Gynecological Cancer Patients<br>6: 6 female, 0 male<br>(66 yrs; 56–85 yrs)<br>0 observation, 6 ongoing therapy	1/2/0/3	54d; 20–84d	66.7%	100%	138.9 (18.9–250.0)
	ENT Cancer Patients<br>9: 4 female, 5 male<br>(68 yrs, 62–90 yrs)<br>1 observation, 8 ongoing therapy	2/0/0/7	50d; 21–81d	77.8%	77.8%	173.0 (0.39–250.0)
	Patients with other cancers<br>12: 7 female, 5 male<br>(63 yrs; 33–85 yrs)<br>4 observation, 8 ongoing therapy	5/5/0/2	47d; 25–102d	50%	58.3%	243.5 (4.62–250.0)
**Vaccinated hematologic patients**	*Myeloid Disorders*<br>36: 13 female, 23 male<br>(77 yrs; 37–89 yrs)<br>6 observation, 30 ongoing therapy	7/21/0/8	48d; 11–107d	69.4%	58.3%	19.05 (5.42–247.0)
	Lymphoid Disorders<br>69: 36 female, 33 male<br>(71 yrs; 25–88 yrs)<br>34 observation, 35 ongoing therapy	14/21/0/33	49d; 10–130d	69.6%	49.3%	14.0 (0.39–78.8)
	Other Disorders (Aplastic Anemia, Hemochromatosis)<br>2: 0 female, 2 male<br>(75, 76 yrs)<br>0 observation, 2 ongoing therapy	0/2/0/0	43d; 38–48d	100%	100%	250.0 (250.0–250.0)

Yrs: years; BAU/ml: Binding Antibody Units/Milliliter; CI: Confidence Interval; SCLC: Small Cell Lung Cancer; NSCLC: Non-Small Cell Lung Cancer; ENT: Ear-Nose-Throat.

Table S6. Patient characteristics and BAU/mL values of all vaccinated hematologic patients with lymphoid disorders, grouped by cell of origin of the disease.

	Patient characteristics (Age: Median; Minimum –Maximum)	Vaccine Type (Vaxzevria®/Comirnaty®/COVID-19 Vaccine Janssen®/COVID-19 Vaccine Moderna®)	Ab-Analysis After First Vaccination (Median; Minimum–Maximum)	Vaccination Scheme Completed	Ab-Levels ≥15 BAU/mL	BAU/mL Median (95% CI of Median)
**Vaccinated hematologic patients with Lymphoid Disorders**	*B-Cell Disorders*					
	41: 20 female, 21 male (71 yrs; 31–88 yrs) 20 observation, 21 ongoing therapy	11/10/0/20	47d; 10–84d	65.9%	31.7%	0.39 (0.39–14.0)
	Plasma Cell Disorders					
	21: 11 female, 10 male (72 yrs, 40–88 yrs) 7 observation, 14 ongoing therapy	3/7/0/10	50d; 24–130d	81%	81%	250.0 (28.5–250.0)
	Mb. Hodgkin + Other Lymphoid Disorders					
	7: 5 female, 2 male (68 yrs; 25–88 yrs) 7 observation, 0 ongoing therapy	0/4/0/3	61d; 27–88d	71.4%	57.1%	177.0 (0.39–250.0)

Yrs: years; BAU/ml: Binding Antibody Units/Milliliter; CI: Confidence Interval

© 2021 by the authors. Licensee MDPI, Basel, Switzerland. This article is an open access article distributed under the terms and conditions of the Creative Commons Attribution (CC BY) license (http://creativecommons.org/licenses/by/4.0/).
